# DAILY EMOTIONS AND STRESS: AGE CHANGES AND DIFFERENCES

**DOI:** 10.1093/geroni/igz038.2136

**Published:** 2019-11-08

**Authors:** Jessica Blaxton, Cindy Bergeman, Lijuan (Peggy) Wang

**Affiliations:** 1 Fontbonne University, St. Louis, Missouri, United States; 2 University of Notre Dame, Notre Dame, Indiana, United States

## Abstract

Developmental processes differ between individuals (interindividual differences), fluctuate within them on a short-term basis (intraindividual variability), and change over time on a longer-term basis (intraindividual change; Nesselroade, 1991). We situate the relationship between stress and emotions in this process-oriented perspective by examining how the daily relationship between stress and negative affect (NA) as well as stress and positive affect (PA) change over time, while considering cross-sectional age and stress differences. Participants (N = 966) completed daily questionnaires assessing stress, NA, and PA. Three-level multi-level models depicted how cross-sectional age, within-person age changes, and global stress differences impact the daily stress-affect relationship. Findings illustrate that cross-sectional age and the aging process uniquely buffer the stress-NA relationship whereas global stress exacerbates it. Furthermore, older adults as well as adults with low global stress experience a weaker relationship between daily stress and PA as they age, but midlife adults and adults with high global stress experience a stronger relationship. These results depict differences in aging trajectories for both midlife and older adults and thus inform intervention and preventative care strategies aimed toward promoting emotional well-being, suggesting that targeting these strategies at the daily level can promote better stress regulation. Furthermore, we see that midlife adults and adults with greater global stress perceptions are most in need of these interventions, and encouraging these adults to maintain PA in the face of daily stress can be particularly beneficial.

